# Aerosolized Thyroid Hormone Prevents Radiation Induced Lung Fibrosis

**DOI:** 10.3389/fonc.2020.528686

**Published:** 2020-09-15

**Authors:** Long Li, Xiaoqi Nie, Minxiao Yi, Wan Qin, Fang Li, Bili Wu, Xianglin Yuan

**Affiliations:** Department of Oncology, Tongji Hospital, Huazhong University of Science and Technology, Wuhan, China

**Keywords:** radiation induced lung fibrosis, aerosolized, thyroid hormone, TGF-β1, macrophage, hypothyroidism

## Abstract

Radiation induced lung fibrosis (RILF) is a common late complication after radiotherapy without effective treatment. Thyroid hormone (TH) is known to reverse bleomycin-induced pulmonary fibrosis in recent study. We therefore sought to examine TH effect in RILF. Aerosolized TH delivery prevented pulmonary fibrosis according to either micro-computed tomography scans or histological evaluations, without significant changes in serum THs in a murine model of RILF by attenuating TGF-β1 and phosphorylated Smad2/3 expressions and reducing the accumulation of M2-like macrophages. Furthermore, hypothyroidism was significantly correlated with RILF in a retrospectively analyzed data from nasopharyngeal carcinoma patients treated by intensity-modulated radiation therapy with a median follow-up time of 25.5 months. Together, aerosolized TH may prevent RILF by inhibiting the TGF-β1/SMADs signaling pathway.

## Introduction

Radiotherapy is widely used in tumor treatment. More than 50 percent of malignant cancer patients require radiation therapy for both curative and palliative purposes (1). However, the application of radiotherapy is limited by the radiation-induced lung injury (RILI), which is a common complication after radiotherapy of thoracic malignant tumors. RILI often causes early radiation pneumonitis and late-onset radiation induced lung fibrosis (RILF) usually occurs in 1 year after radiotherapy (2). The incidence of symptomatic RILF in patients receiving thoracic radiotherapy is about 5–24%, and higher in patients with subclinical damage (3). Because effective treatments are lacking, RILF has an adverse impact on the quality of life of suffering patients, causing cough, shortness of breath, fever, progressive respiratory dysfunction, or even death (4–6). Currently, corticosteroids are commonly used in the clinic for radiation induced lung injury. Several prevention strategies have shown certain promising effects in murine models of RILF, including treatments with amifostine, ACE inhibitors, angiotensin II receptor inhibitors, pentoxifylline as well as with inhibitors of PDGF, VEGF, FGF, TGF-β1, and Cox-2, but firm clinical evidence is lacking (7–9).

Thyroid hormone is one of the most important hormones that regulate energy metabolism. The levels of active triiodothyronine and its precursor thyroxine (T4) are mainly regulated by DIO2 in cells and tissues (10). There is growing evidence of the correlation between thyroid function and fibrosis disease in patients. Grazinao (11) found that patients with idiopathic retroperitoneal fibrosis (IRF) had a higher risk of hypothyroidism than controls (OR = 3.56, 95%CI 1.48–8.59, and *P* = 0.004). Nearly a quarter of IRF patients received L-thyroxine at the end of the follow-up (median, 45 month), but only 3% of controls needed treatment in the same period. The incidence of hypothyroidism in idiopathic pulmonary fibrosis (IPF) was 16.8%, compared with 7.1% in control group; multivariate analysis showed that hypothyroidism was an independent predictor of death risk in IPF patients (12). Clinical data from a large sample showed that hypothyroidism significantly correlated with non-alcoholic fatty liver fibrosis (OR = 2.23, 95%CI 1.18–4.23, and *P* = 0.014) (13). Hypothyroidism also plays an important role in myocardial fibrosis (14) and diabetic nephropathy (15).

Furthermore, recent studies have shown that TH may have a therapeutic effect in acute lung injury (16, 17). Yu et al. (18) demonstrated that TH significantly attenuated adverse signs in a mouse model of bleomycin-induced pulmonary fibrosis by restoring epithelial mitochondrial function. TH efficacy was higher than that of pirfenidone and nintedanib, which are currently approved for IPF by the US Food and Drug Administration. That study suggested that in contrast to the expensive drugs mentioned above, TH may have broad application prospects in IPF. However, the role of TH in RILF has not been investigated so far.

It is generally accepted that TGF-β1 (19, 20) and the polarization of M2 phenotype macrophages (21, 22) play critical roles in multiple organ fibrosis as well as in RILF. Therefore, inhibition of the TGF-β1 signaling pathway may be a plausible strategy to alleviate RILF. It has been reported that TH significantly reduced liver fibrosis and skin fibrosis in mice by inhibiting transcriptional activation evoked by TGF-β/SMAD (23). Based on this evidence, we explored potential protective effects of TH in RILF in the present study.

## Materials and Methods

### Animals and Radiation

Prednisone (PDN, HY-B0214) and 3,3′,5-triiodo-L-thyronine (T3, HY-A0070A) were purchased from MedChemExpress (Monmouth Junction, NJ, United States) and formulated in dimethyl sulfoxide solution. RILF was modeled in female C57BL/6 mice because they were likely to develop lung fibrosis (24). A total of 60 female C57BL/6J mice (Experimental Animal Center of Hubei province, China) aged 6–8 weeks were randomly assigned to five groups: control group (*n* = 12), aerosolized T3 group (*n* = 12), radiation treatment (RT) group (*n* = 12), RT + T3 group (*n* = 12), and RT + PDN (*n* = 12). Drug delivery of T3 or PDN was started on the first day of RT and continued daily for 1 month after RT. Each time, six mice were simultaneously exposed to a nebulizer chamber where T3 suspension was aerosolized at a dose of 40 μg/kg in 6 mL of phosphate buffered saline by an ordinary aerosol nebulizer (Omron) until atomization stopped (18). PDN was given intraperitoneally at 5 mg/kg. Mice developed pulmonary fibrosis after RT at a single dose of 16 Gy, as previously described (25). Mice were maintained in the specific pathogen-free animal facility of the Huazhong University of Science and Technology.

### Micro-CT Scan

At weeks 6, 16, and 25 after radiation, three mice per group were randomly selected and sacrificed for thorax CT with a micro-CT scanner (Skyscan 1176, Bruker Inc, Billerica, MA, United States). The obtained CT images were imported into RadiAnt DICOM Viewer 3.4.1^1^. Lung slice image analysis was performed in the most typical slice that exhibited pulmonary fibrosis features. Four regions per slice were selected as measurement points: anterior and posterior in right and left lungs. Quantitative lung density of one mouse was represented as the mean Hounsfield unit (HU) value ± standard error of the mean (SEM).

### Lung Histology and Fibrosis Score

Lung histological analysis was conducted as previously described (25). Briefly, three mice per group were euthanized for histology on the days of micro-CT chest scans. 25 weeks post RT, all remaining mice were killed by cervical dislocation. The left lungs were fixed in 4% paraformaldehyde for 24 h, embedded in paraffin, then cut into 5 μm thick slices and stained with hematoxylin-eosin stain and Masson’s trichrome stain for collagen deposition.

The degree of pulmonary fibrosis was assessed by the modified Ashcroft score (26) on a range from 0 to 8 by examining five random microscopic fields at 200 × magnification. The final fibrosis degree was determined by an average score of all fields.

Expression of proteins was examined by immunohistochemistry (IHC) using staining with antibodies against αSMA (1:100, 14395-1-AP, Proteintech, Wuhan, China), F4/80 (1:400, ab111101, Abcam, United Kingdom), CD163 (1:200, ab182422, Abcam), iNOS (1:400, ab15323, Abcam), CD206 (1:200, ab64693, Abcam), and DIO2 (1:200, 26513-1-AP, Proteintech) according to previously described standard procedures (25). IHC results were semi-quantitatively analyzed. In brief, each sample was scored by combining immunoreactive signal intensity (negative = 0, weak = 1, moderate = 2, and strong = 3) and the percentage of positively stained cells (<10% positive cells = 0, 10%–30% positive cells = 1, 30%–50% positive cells = 2, and >50% positive cells = 3). All slides were individually analyzed by two pathologists, using a light microscope. All histological samples were randomly numbered in a blinded fashion to avoid observer bias.

### Human Tissue Samples

Human lung samples were collected from the Department of Pathology of the Tongji Hospital, guided by Ethics Board at the hospital without the need for specific consent. Lung samples were obtained from pulmonary malignancy patients who underwent surgery more than 6 months after radiotherapy. Surgical lung specimens from patients that did not receive radiotherapy at the same period were included as control. All patients were operated at the Department of Thoracic Surgery in our hospital.

### Patient Population and Follow-Up

In order to explore the role of hypothyroidism in RILF, we retrospectively analyzed data from nasopharyngeal carcinoma patients (NPC) treated by intensity-modulated radiation therapy (IMRT) at the Tongji Hospital of the Huazhong University of Science and Technology between January 2010 and December 2018, for thyroid dysfunction is prevalent after radiotherapy in NPC. The bilateral upper lungs were delineated on computed tomography (CT) scans, and lung doses were calculated. Eligible cases had to meet the following inclusion criteria: confirmed NPC without lung metastasis was present before radiotherapy; IMRT was conducted in our hospital, thus complete information was available; thyroid function was examined before and after IMRT; patients with hypothyroidism before IMRT were also included in the analysis; patients had a series of chest CT scans in the follow-up every 2–3 months within 2 years after treatment, and every 3–6 months later, finally annually after 5 years. We excluded subjects who had no irradiation dose in lungs, were diagnosed with connective tissue diseases, or were followed for less than 3 months. We restaged all patients using the 7th American Joint Committee on Cancer staging system. RILI was confirmed according to Common Terminology Criteria for Adverse Events (version 4.0).

### Serum TH Assay

Total serum TH in NPC patients, including thyroid-stimulating hormone (TSH), free triiodothyronine (fT3), and free thyroxine (fT4) were measured in the Laboratory Department of our hospital by using the electrochemiluminescence immunoassay. The normal values for TSH, fT3, and fT4 were considered to be 0.35–4.94 μIU/mL, 1.71–3.71 pg/mL, and 0.7–1.48 ng/dL, respectively. The presence of abnormally elevated TSH in the serum was defined as hypothyroidism, with or without decreases in fT4.

Serum samples were prepared from mouse blood collected from eyelids using BD vacutainers on the days of micro-CT chest scans. In the remaining mice, serum was obtained at week 25 after RT using the same method. Total serum T3, T4, and TSH levels were measured using a T3 (ELK1339, ELK Biotechnology, Wuhan, China), T4 (ELK1204, ELK Biotechnology), and TSH (ELK2284, ELK Biotechnology) ELISA kits, following the manufacturers’ protocols.

### Hydroxyproline Assay

Right lung hydroxyproline level was analyzed with a hydroxyproline colorimetric assay kit (A030-2, Nanjing Jiancheng Bioengineering Institute, Nanjing, China) to assess collagen content as previously described (25).

### Western Blot Analysis

Total protein was extracted from mouse left lung tissue, and its concentration was determined using the bicinchoninic acid assay. Then the constituent proteins were separated by electrophoresis in a 10% sodium dodecyl sulfate polyacrylamide gel, transferred to polyvinylidene fluoride membranes (Millipore, Billerica, MA, United States), covered with 5% milk at room temperature for 1 h, and incubated overnight with appropriate primary antibodies diluted in 0.1% Tween 20 (TBST) at 4°C. Primary antibodies against the following proteins were used: SMAD2/3 (8685T, Cell Signaling Technology, Beverly, MA, United States), p-SMAD2/3 (8828S, Cell Signaling Technology), TGF-β1 (3711S, Cell Signaling Technology); GAPDH (AC002, Abclonal, Wuhan, China); αSMA (14395-1-AP, Proteintech), collagen I (14695-1-AP, Proteintech), and PAI-1 (TA504056S, Origene, Maryland). After washing with TBST, the membranes were incubated with anti-rabbit or anti-mouse IgG horseradish peroxidase conjugated antibody (Cell Signaling Technology) for 1 h at room temperature. The protein bands were visualized using SuperSignale West Pico plus Chemiluminescent Substrate (Thermo Fisher Scientifice, Waltham, MA, United States).

### Statistics

For the analysis of patient data, univariate analysis was performed by the chi-square test, Fisher’s exact test, or Student’s *t*-test to find possible risk factors associated with RILF. One-way analysis of variance (ANOVA) was used to reveal whether the grouping factor (treatment) differentially affected the results in more than three groups. Significant variables in univariate analysis were included into multivariate analysis with binary logistic regression model. Comparisons between the experimental groups for lung density, hydroxyproline content, fibrosis score, and protein expression levels were performed by ANOVA or Student’s *t*-test using Prism 6.01 (GraphPad Software, San Diego, CA, United States) or SPSS 19.0 (IBM Corp, Armonk, NY, United States). All statistical analyses were conducted with a significance level of α = 0.05 (*P* < 0.05).

## Results

### Pulmonary Fibrosis Development in RT Treated Murine Model

**FIGURE 1:**
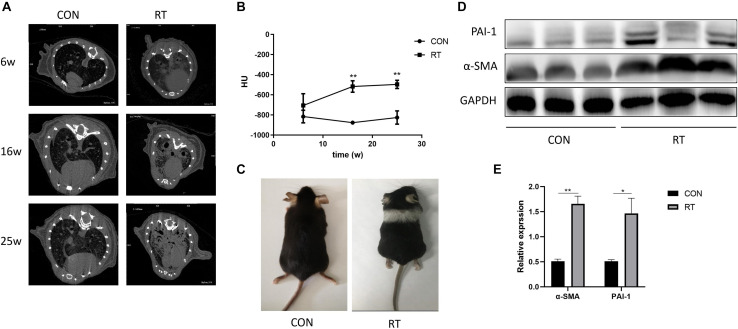
Establishment and confirmation of radiation-induced lung fibrosis in a murine model. **(A)** Representative images of micro-CT scan for lung density at weeks (W) 6, 16, and 25 after irradiation in control or RT group. **(B)** Lung density evaluated by Hounsfield units (HU) presented as the mean ± SEM in radiation-treated and control C57BL/6J mice over time (*n* = 3); *P < 0.05. **(C)** Photograph of a representative mouse at week 16 after radiation treatment. **(D,E)** The expression levels of α-SMA and PAI-1 in treated or control groups at week 16 was determined by western blotting. **P < 0.01.

Micro-CT scans performed at three time points after RT revealed increased number of diffuse and patchy shadows in the RT group. A trend of increase in lung density was observed after RT, which turned to be significantly consolidated at week 16 and gradually stabilized over 6 months, suggesting the development of RILF (Figures 1A,B). The irradiated field of mouse hair slowly turned gray during the post-irradiation period (Figure 1C). This indicated that our irradiated area coincided with the lungs of the mice. The western blot assay indicated that α-SMA, a myofibroblast activation marker that indicates fibrosis severity, and PAI-1 expressions increased significantly in lung tissue at week 16 after RT (Figures 1D,E). All these results suggested obvious fibrotic changes in lung tissues by week 16 post RT.

### Attenuation of RILF Development in Mouse Lungs by TH

**FIGURE 2:**
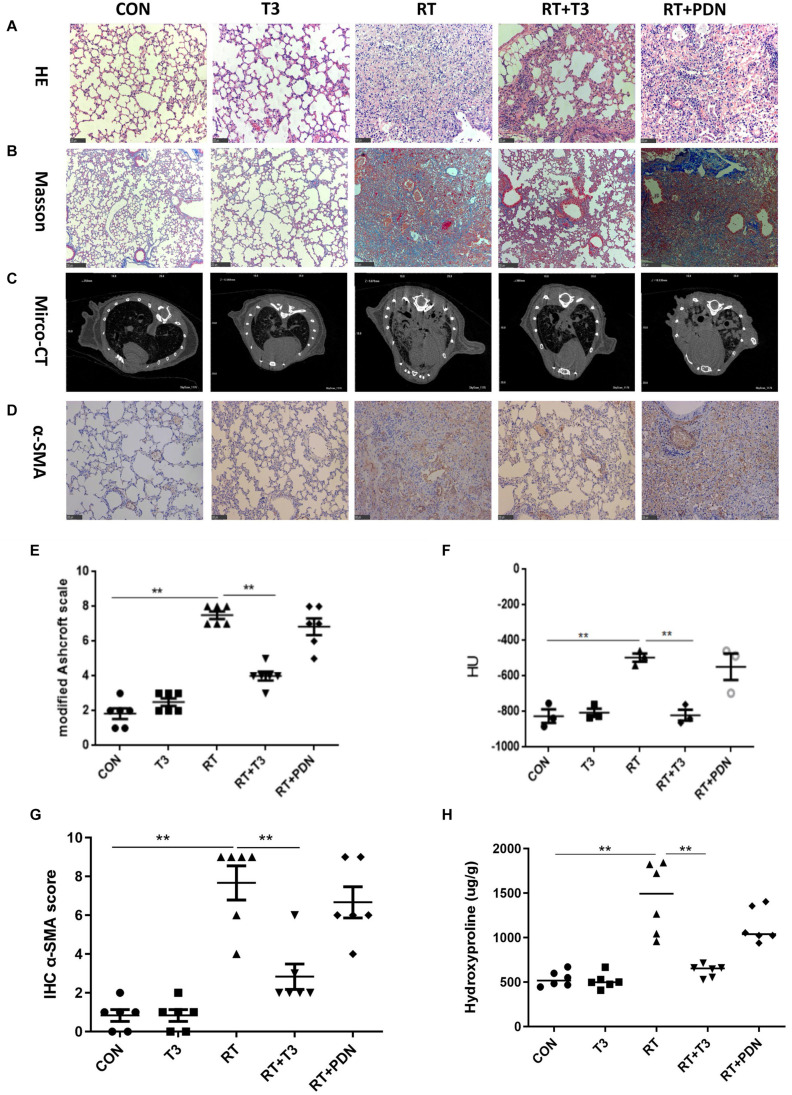
Aerosolized T3 prevents pulmonary fibrosis post radiation treatment in mice. **(A,B)** Representative images of hematoxylin/eosin (200×; scale bar = 100 μm) and Masson’s trichrome staining (100×; scale bar = 250 μm) from each group at 25 weeks after radiation treatment. **(C)** Images of representative micro-CT scans obtained at week 25 post radiation treatment in each group. **(D)** Representative immunohistochemistry staining images with α-SMA protein expression (200×; scale bar = 100 μm) in lung tissues in each group at the end of the experiment. **(E)** Grading of pulmonary fibrosis evaluated blindly by the modified previously described Ashcroft Scale at week 25 post radiation treatment. **(F)** Lung density values evaluated quantitatively by Hounsfield units derived from micro-CT scans at week 25 post irradiation (*n* = 3 for each individual group). **(G)** Immunohistochemistry scores for lung histopathological changes calculated using a semi-quantitative scoring system. **(H)** Collagen deposition in mouse right lung tissue assessed by measuring hydroxyproline content in each group at the end of the experiment. Data are expressed as the mean ± SEM, *P < 0.05, **P < 0.01. There were six mice in each group, except where indicated otherwise.

To test the potential radioprotective effect of TH in RILF, micro-CT and histological analyses of lung samples were performed under the light microscope at 6 months post RT in each experimental group. Manifestations such as the presence of alveolar wall thickening, fibrotic nodules, and destruction of alveolar structures after irradiation were less pronounced in the RT + T3 group but not in the RT + PDN group, as compared to the parameters in RT group according to hematoxylin and eosin staining (Figure 2A) and modified Ashcroft scale (*P* < 0.01, Figure 2E). No specific reduction of blue collagen deposition was observed by using Masson’s trichrome stain (Figure 2B) at the end point of the experiment in RT + PDN group compared to the level of blue collagen in RT group. In contrast, we found that lung hydroxyproline content was dramatically decreased in RT + T3 group (Figure 2B; *P* < 0.05, Figure 2H) and α-SMA expression was markedly reduced (Figure 2D; *P* < 0.01, Figure 2G) compared to the values in RT group. Additionally, we observed appearance of abnormal morphological changes of patchy shadows, ground-glass opacity, and consolidation of large areas of lung tissues (Figure 2C) in RT and RT + PDN groups. However, the treatment of PDN could not inhibit the formation of pulmonary fibrosis caused by RT. We further quantitatively evaluated pulmonary fibrosis by estimating lung density using HU values. A reduction of approximately 324 HU (Figure 2F) was observed in RT + T3 group compared to the value in RT group at 25 weeks post RT.

### Lack of Change in Serum TH Levels After Pulmonary Aerosolized T3 Delivery

**FIGURE 3:**
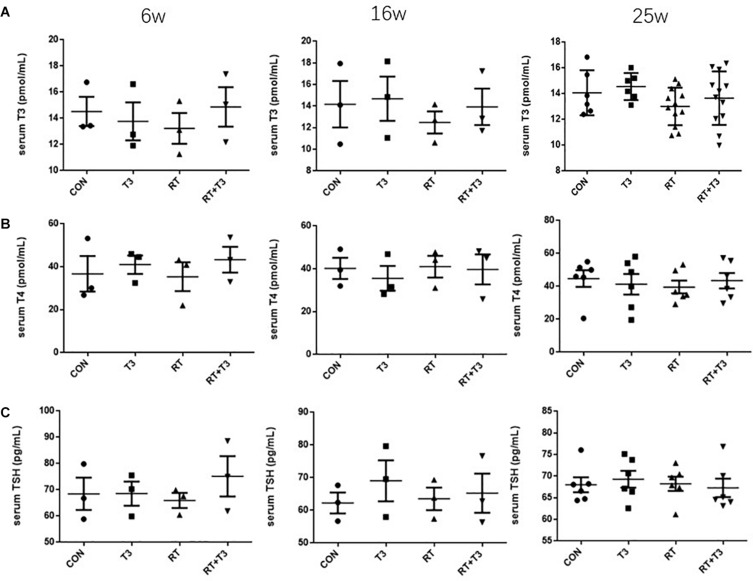
Serum levels of thyroid hormones in mice at different time points. **(A–C)** Serum T3 (pmol/mL), T4 (pmol/mL), and TSH (pg/mL) levels in each experimental group at weeks 6, 16, and 25 after radiation treatment. No significant effect of time on concentrations was observed. Data are presented as the mean ± SEM. Weeks 6 and 16: *n* = 3; week 25: *n* = 6.

In order to improve drug absorption in the lungs, aerosolized T3 (40 μg/kg) was administered to treated mice. We sought to verify whether this method of administration affected serum thyroid function levels and/or had possible side effects. Serum thyroid function tests were performed at 6, 16, and 25 weeks after radiation scheduled on micro-CT chest scans. There were no significant differences in serum T3, T4, and TSH levels among these groups at those three experimental points (Figures 3A–C). These results suggested that there was no obvious correlation between the improvement of RILF by aerosolized T3 and possible changes in serum TH levels.

### Increased Risk of RILF in Patients With Hypothyroidism

**FIGURE 4:**
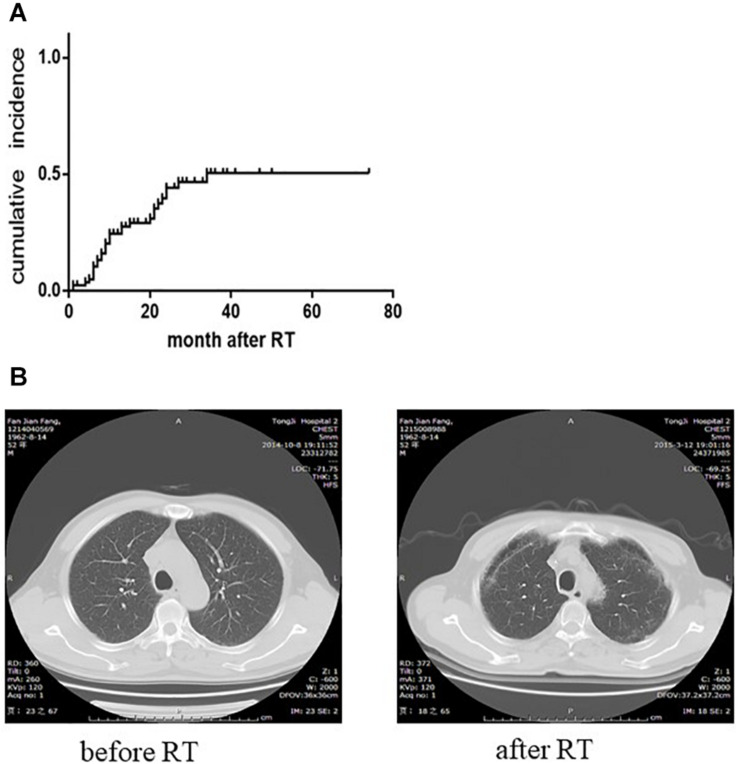
Changes of serum thyroid function and bilateral pulmonary apex in patients with nasopharyngeal carcinoma after radiotherapy. **(A)** The cumulative incidence curve of radiation-induced hypothyroidism (two patients had already suffered from hypothyroidism before radiotherapy). The cumulative incidence was 49.58% at 3 years after radiotherapy. **(B)** CT images of patients before and after radiotherapy. Increased density of apex or superior lobe of lungs is seen on the follow-up CT examination after radiotherapy. Such changes without serious clinical symptoms could be classified as grade 1 radiation-induced lung injury.

Next, we conducted a single-center retrospective clinical case study. NPC patients that were regularly examined for serum TH and had chest CT scans before and after IMRT were included in this investigation. Dosimetric parameters of patients’ lungs were calculated by a physicist. A total of 82 patients from January 2010 to August 2018 met the inclusion criteria. Two patients had already suffered from decreased thyroid function at the beginning of radiotherapy. Hypothyroidism is mainly caused by significantly elevated TSH. Of the 82 subjects, 61 males and 21 females, 38.75% of the patients developed hypothyroidism (Figure 4A) and 22.0% (18 patients) had mild RILF (Figure 4B) during the median follow-up time of 25.5 months (2–79 months).

In the univariate analysis, V50 Gy was 16.11 ± 13.59 cm^3^ in the RILI group and 4.29 ± 5.77 cm^3^ in the non-RILI group (*P* = 0.002). At the same time, hypothyroidism [odds ratio (OR) 4.095, 95% confidence interval (CI) 1.349–12.429, *P* = 0.01] and N stage (*P* = 0.018) were also significantly correlated with RILI, respectively, (Table 1). For the N stage, chi-square test showed that N3 stage was significantly associated with RILI (N3/N1: OR 6.60, 95%CI 1.515–28.747, *P* = 0.014; N3/N2: OR 5.20, 95%CI 1.181–22.891, *P* = 0.047), while N2 had no statistical significance compared with N1 (OR 1.269, 95%CI 0.366–4.398, *P* = 0.707). Finally, multivariate logistic regression analysis model found that V50 Gy (OR 1.173, 95% CI 1.077–1.278, *P* < 0.001) and hypothyroidism (OR 6.137, 95%CI 1.448–26.002, *P* = 0.014) were significantly associated with RILI (Table 2).

**TABLE 1:** Univariate analysis of radiation-induced lung injury risk factors.

Variable	RILI	non-RILI	*P* value
Gender			0.709
Male	14	47	
Female	4	17	
Year	48.6 ± 8.10	46.1 ± 9.60	0.333
T stage			0.481
T1-2	6	16	
T3-4	12	48	
N stage			**0.018**
N1	6	33	
N2	6	26	
N3	6	5	
M stage			0.22
M0	17	64	
M1	1	0	
V50 Gy, cm^3^	16.11 ± 13.59	4.29 ± 5.77	**0.002**
Induction chemotherapy			0.108
NO	0	10	
YES	18	54	
Concurrent chemotherapy			0.678
NO	1	7	
YES	17	57	
Hypothyroidism			**0.01**
NO	6	43	
YES	12	21	

*Values are expressed as number or mean ± SD. Variables that were significantly altered (*P* < 0.05) are highlighted. RILI, radiation induced lung injury; V50 Gy, normal lung volume that received radiation dose > 50 Gy.*

**TABLE 2:** Multivariate analysis of radiation-induced lung injury risk factors.

Variable	OR	95%CI	*P* value
Hypothyroidism	6.137	1.448–26.002	0.014
V50	1.173	1.077–1.278	<0.001

*OR, odds ratio and CI, confidence interval.*

### Higher Expression of DIO2 in Human RILF Tissues and Inhibition of TGF-β1 Signaling in the Lung by TH

**FIGURE 5:**
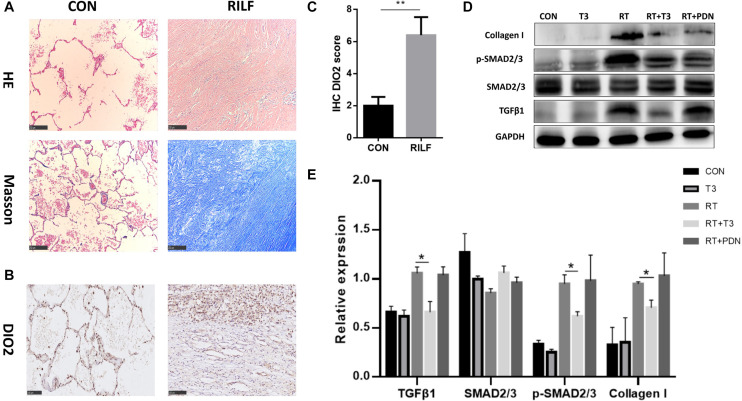
Increased DIO2 expression in lung tissue after radiation treatment and inhibition of the TGF-β1 signaling pathway by thyroid hormone. **(A)** Representative images of hematoxylin/eosin and Masson’s trichrome staining (100×; scale bar = 250 μm) from treated (*n* = 5) and control (*n* = 5) patients. Absence of alveolar structures and apparent collagen deposition can be seen in the irradiated lung tissue. Representative immunohistochemistry staining **(B)** and immunohistochemistry scores **(C)** for DIO2 (200×; scale bar = 100 μm) in human lung tissue samples (*n* = 5) showing DIO2 expression in the lung affected by radiation-induced lung fibrosis (left) and in control lung (right). **(D,E)** Western blots of profibrotic growth proteins (collagen I, TGF-β1) and p-Smad2/3, a downstream protein in the TGF-β1 signaling pathway, in mouse lung tissues of each group at 25 weeks post radiation treatment are shown. *P < 0.05; **P < 0.01.

Irradiated lung tissue was acquired from the surgical samples of five patients treated between 2011 and 2018 in our hospital. They were four squamous lung cancers and one lung metastases from colon carcinoma, who had relapsed *in situ* after radiation therapy. They all had received preoperative radiation treatment with a total dose ranging from 50 to 66 Gy between year 2008 and 2017. Untreated control lung samples were obtained from another five patients that did not receive any radiotherapy. In contrast to control samples, samples from patients with RILF displayed thickening of the alveolar septum or loss of alveolar structure and deposition of collagen fibers (Figure 5A). Considering the established role of iodothyronine deiodinase in T4 to active T3 conversion in tissues, we investigated the expression of DIO2 gene in order to verify the relationship between TH and RILF. Interestingly, DIO2 was highly expressed in epithelial cells within fibrotic regions of irradiated human lung tissue compared to its low-level control group (Figures 5B,C), suggesting that locally increased T3 concentration may improve energy metabolism of alveolar epithelial cells in the stressed fibrotic lung. Given the very limited number of human RILF tissue samples, this result implies a potentially protective role of TH on RILF.

In order to investigate the mechanisms whereby T3 exerts a protective effect on RILF, we evaluated the expression of pro-fibrotic growth factors in the lung tissues of mice using western blotting. Collagen I and TGF-β1 were found to be highly expressed in the lungs of RT and RT + PDN groups. In contrast, the expression of the principal pro-fibrotic factor TGF-β1 was decreased in sham RT and RT + T3 groups. In addition, western blotting also demonstrated that phosphorylated Smad2/3 levels were reduced in RT + T3 group (Figures 5D,E). These results suggested that T3 likely mitigated RILF by inhibiting expression of TGF-β1 and its downstream signaling molecules.

### M2 but Not M1-Like Macrophages Accumulate in RILF

**FIGURE 6:**
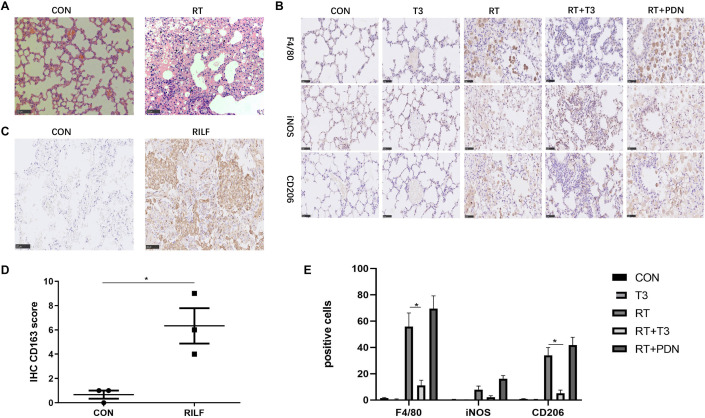
Macrophage accumulation in the lungs of mice and humans after thoracic radiotherapy. **(A)** Representative images of macrophage in mice lung 25 weeks post irradiation compared to controls in HE staining. arrows indicated macrophages (200×; scale bar = 100 μm). **(B)** Representative images of total, M1 and M2 macrophages in lung tissues of each group identified by F4/80, iNOS, and CD206, respectively, using immunohistochemistry (400×; scale bar = 50 μm). **(C)** Representative immunohistochemistry images of CD163 positive macrophage in lung tissues (200×; scale bar = 100 μm). **(D)** Imimmunohistochemistry assessment for macrophage quantification in lungs of control and irradiation patients (*n* = 3 per group). **(E)** The numbers of F4/80, iNOS, and CD206 positive macrophages were counted at high magnification field and take the average of three times (*n* = 3 per group), data are expressed as the mean ± SEM, *P < 0.05.

We explored the role of macrophages in clinical and preclinical animal levels. Compared with the control group, scattered macrophages could be found in the pulmonary fibrosis area of mice at 25 weeks after irradiation, which had the morphological characteristics of nuclear deviation and relatively large cell volume (Figure 6A). F4/80 (macrophage marker) positive cells were observed in pulmonary fibrosis tissues induced by irradiation than that of sham irradiation controls (*P* < 0.01, Figures 6B,E). Notably, in the lung tissues of RT + T3 group, but not RT + PDN group, the decrease of F4/80 positive macrophage proportion was detected along with the remission of pulmonary fibrosis (*P* < 0.05, Figures 6B,E). An obvious increase of M2-like macrophages (CD206 positive cells) were predominantly located in lung fibrosis tissues. Moreover, M2-like macrophages were significantly reduced in RT + T3 group (*P* < 0.05, Figure 6E). In contrast, there was no significant change in the proportion of M1-like macrophages (iNOS positive cells) in each group (Figure 6E). We then also assessed the accumulation of M2-like macrophages in irradiated human lung tissues. Compared to non-irradiated control lung tissues, M2-like macrophages (CD163 positive cells) were significantly increased in human pulmonary fibrotic area (*P* < 0.05, Figures 6C,D). These results suggest that M2 rather than M1-like macrophages are associated with RILF, and that aerosolized T3 attenuates RILF in mice accompanied by a decrease in M2-like macrophages in the fibrosis tissue.

## Discussion

Radiation induced lung fibrosis is a common complication in the management of radiotherapy and it seriously affects patients’ quality of life. To date, however, there is still no viable therapeutic strategy for RILF, and its mechanism remains unclear. With the growing incidence and mortality rate of lung cancer worldwide (27), therapies that prevent RILF represent an unmet clinical need. In the current study, we have demonstrated a therapeutic effect of aerosolized T3 treatment in a rodent experiment model during the post RT phase. Secondly, we showed that the antifibrotic effect of T3 does not require increased serum levels of T3, T4, or TSH, suggesting that aerosolized delivery may be effective without side effects of iatrogenically elevated thyroid hormones. Thirdly, we found that hypothyroidism increases the risk of RILF in NPC patients; moreover, M2-like macrophages were associated with RILF. Considering these results, we suggest the delivery of aerosolized T3 as a new potential treatment strategy for RILF attenuation.

Thyroid hormone regulates diverse biological processes, from growth to metabolism, and is critically important for nearly all tissues (28–30). Both T4 and T3 can be deiodinated either into the active form by DIO1 and DIO2 or into the inactive form — by DIO3 (31). DIO2 plays a major role in the synthesis of biologically active TH. Increased expression of DIO2 in tissues may reflect either a lack of TH or the need for increased metabolism. Previous studies have shown that hypothyroidism is associated with poor prognosis in many critical diseases, including heart failure (32), non-alcoholic fatty liver disease (33), chronic kidney disease (34), and lung disease (28). Hypothyroid mice suffered more severe lung injury than those with normal serum TH levels in a mouse model of ventilator-induced lung injury, and administration of T3 reduced chemokine and cytokine levels in *Dio2* knockout mice (16).

Our results showed that the relationship between both the expression of DIO2 protein as well as TH levels and RILF was consistent with the above results, indicating that there is a correlation between RILF and TH. It is common to find pulmonary shadows in follow-up CT image examinations in some NPC patients due to upper lungs being exposed to the radiation field, which could also be classified as RILI. According to two population-based studies, the incidence of radiation-induced hypothyroidism varied from 27 to 70% in patients that received a dose of 7.5–40 Gy (35, 36). The incidence of hypothyroidism induced by radiation was 38.75% during the follow-up in our study. All patients observed in this cohort had subclinical hypothyroidism. The reasons for this finding likely include insufficient follow-up duration in a small sample and wide use of IMRT in our hospital (37, 38). Bhandare’s study (39) reported that the median latency of clinical hypothyroidism was 4.8 years. RILF occurred in 18 (22.0%) of 82 patients from our cohort during a median follow-up of 25.5 months (range 2–79 months). The proportion of RILF in subjects with hypothyroidism was obviously higher than that in subjects with normal thyroid function, and hypothyroidism was significantly associated with RILF in multivariate logistic regression analysis model. This finding may help understanding the mechanisms of RILF occurrence.

The pathological mechanism of hypothyroidism leading to RILF remains unclear. One possible link may lie in thyroid transcription factor-1 (TTF-1). It plays an important role in the differentiation and formation of both thyroid and lung. Increased expression of TTF-1 was found in some thyroiditis patients (40). In the lung, TTF-1 regulates the differentiation of alveolar epithelial cells and the expression of alveolar surfactant protein, which is very important to maintain alveolar ventilation function and repair lung injury (41). Another possible link maybe related to the biological functions of thyroid hormone, which is not only an important in regulating human endocrine metabolism, but also affects mitochondrial function and transformation. Mitochondrial damage contributes to the development of RILF.

Treatment with TH, an old but probably underused drug, may be utilized in cases with pathologies other than thyroid dysfunction. Some synthetic TH mimetics have shown encouraging results in the experimental treatment of obesity, dyslipidemia, and liver cancer (42). T3 also could alleviate the pulmonary fibrosis in TGF transgenic mice, but the mechanism is not fully explained (18). Recent studies showed that TH attenuated skin and pulmonary fibrosis induced by bleomycin and liver fibrosis caused by carbon tetrachloride in mouse models. These actions may be explained by TH effects on mitochondrial biogenesis and inhibition of TGF-β1-dependent transcription (18, 23). TGF-β1 plays a critical role in profibrotic signals: about 80% of the proteins encoded by genes dysregulated in pulmonary tissues from IPF patients have been reported to be associated with TGF-β1 signaling pathway (43). RILF is similar to other forms of lung fibrosis, especially IPF. Aerosolized TH treatment significantly reduced expression of profibrotic growth proteins, including collagen I, PAI-1 and TGF-β1, whereas no such down-regulation was observed in the RT + PDN group in our study. We concluded that PDN did not inhibit the elevation of TGF-β1 and thus had no anti-fibrotic effect previously suggested by Arata et al. (44). Furthermore, we also consistently found that expression level of phosphorylated Smad2/3, an important transcription factor downstream of the TGF-β1 pathway, was significantly decreased in TH treatment group.

A recent study showed that M2-like tissue-infiltrating macrophages played an important role in RILF (21), but the relationship between local or recruited alveolar macrophages and RILF is still worthy of further study (45). Alveolar macrophages are highly heterogeneous. M1 like macrophages mediate resistance to pathogens, while M2 like macrophages have anti-inflammatory and repair functions (46). T3 can promote the polarization of mouse bone marrow-derived monocytes to M1 macrophages phenotype and inhibit activated M2 macrophage phenotype (47). Tumor infiltrating myeloid-derived cells secrete high levels of TGFβ, and up-regulate CD206 expression (48). In the pulmonary fibrosis model induced by TGF-β1, reduced pulmonary M2 macrophages had a significant anti-fibrosis effect (49). A study suggested that the development of RILF may depend on TGFβ to promote the transformation of macrophages into M2 phenotype (50). However, glucocorticoids can reduce the number of M1 phenotype differentiation of macrophages (51) and have the ability to promote the activation of M2 phenotype macrophages (52). In our study, we also found that the M2 macrophages in lung tissues did not decrease, and even showed a trend of increasing in RT + PDN group. This may partly explain the role of thyroid hormone in alleviating RILF, which may be associated with TGFβ1 and macrophages.

Our study had some limitations. First, our clinical data were obtained from a retrospective analysis of a small size cohort that did not have very long follow-up. Secondly, we included into the analysis the patients that did not receive thoracic radiotherapy and had no advanced RILF. Thirdly, several questions related to the mechanism of TH involvement were not studied. For example, we mainly focused on the DIO2, whereas other deiodinases were not detected, and the mechanism of the role of DIO2 in the development of fibrosis after irradiation need to be further explored. Furthermore, the mechanism underlying inhibitory effects of TH on TGF-β1/SMAD signaling pathway and M2-like macrophage has to be elucidated.

In conclusion, despite recent advances in radiation treatment planning and image-guide radiation therapy, RILF still remains a limiting factor for local tumor control by radiotherapy. To the best of our knowledge, our study for the first time demonstrated that hypothyroidism maybe associated with an increased risk of RILF in patients and provided the first evidence that T3 may be a safe therapeutic option to prevent RILF.

## Data Availability Statement

All datasets generated for this study are included in the article/supplementary material.

## Ethics Statement

The studies involving human participants were reviewed and approved by Ethics committee of Tongji hospital, Huazhong University of Science and Technology. The patients/participants provided their written informed consent to participate in this study. The animal study was reviewed and approved by Institutional Animal Care and Use Committee of the Tongji Medical College at the Huazhong University of Science and Technology. Written informed consent was obtained from the individual(s) for the publication of any potentially identifiable images or data included in this manuscript.

## Author Contributions

LL, XN, and MY carried out experiments. LL and XN analyzed data. WQ and XY designed the experiments. LL, FL, and BW wrote the manuscript. All authors had final approval of the submitted and published versions.

## Conflict of Interest

The authors declare that the research was conducted in the absence of any commercial or financial relationships that could be construed as a potential conflict of interest.

## Footnotes

1 http://www.radiantviewer.com/
